# Development of antidrug antibodies against adalimumab maps to variation within the HLA-DR peptide-binding groove

**DOI:** 10.1172/jci.insight.156643

**Published:** 2023-02-22

**Authors:** Teresa Tsakok, Jake Saklatvala, Theo Rispens, Floris C. Loeff, Annick de Vries, Michael H. Allen, Ines A. Barbosa, David Baudry, Tejus Dasandi, Michael Duckworth, Freya Meynell, Alice Russell, Anna Chapman, Sandy McBride, Kevin McKenna, Gayathri Perera, Helen Ramsay, Raakhee Ramesh, Kathleen Sands, Alexa Shipman, the Biomarkers of Systemic Treatment Outcomes in Psoriasis (BSTOP) Study Group, A. David Burden, Christopher E.M. Griffiths, Nick J. Reynolds, Richard B. Warren, Satveer Mahil, Jonathan Barker, Nick Dand, Catherine Smith, Michael A. Simpson

**Affiliations:** 1 Department of Medical and Molecular Genetics and; 2 St John’s Institute of Dermatology, School of Basic & Medical Biosciences, Faculty of Life Sciences & Medicine, King’s College London, London, United Kingdom.; 3 St John’s Institute of Dermatology, Guy’s and St Thomas’ National Health Service Foundation Trust, London, United Kingdom.; 4 Department of Immunopathology, Sanquin Research and Landsteiner Laboratory, Amsterdam, Netherlands.; 5 Biologics Lab, Sanquin Diagnostic Services, Amsterdam, Netherlands.; 6 Department of Dermatology, Queen Elizabeth Hospital, London, United Kingdom.; 7 Department of Dermatology, Royal Free London National Health Service Foundation Trust, London, United Kingdom.; 8 Department of Dermatology, Belfast Health and Social Care Trust, Belfast, United Kingdom.; 9 Department of Dermatology, Chelsea and Westminster Hospital National Health Service Foundation Trust, London, United Kingdom.; 10 Department of Dermatology, Sheffield Teaching Hospitals National Health Service Foundation Trust, Sheffield, United Kingdom.; 11 Department of Dermatology, Sandwell and West Birmingham National Health Service Trust, Birmingham, United Kingdom.; 12 Department of Dermatology, East Kent Hospitals University National Health Service Foundation Trust, Kent, United Kingdom.; 13 Department of Dermatology, Portsmouth Hospitals National Health Service Trust, Portsmouth, United Kingdom.; 14 See Supplemental Acknowledgments for consortium details.; 15 Institute of Infection, Immunity and Inflammation, University of Glasgow, Glasgow, United Kingdom.; 16 Dermatology Centre, Salford Royal National Health Service Foundation Trust, Manchester, United Kingdom.; 17 The University of Manchester, Manchester Academic Health Science Centre, National Institute for Health Research Manchester Biomedical Research Centre, Manchester, United Kingdom.; 18 Department of Dermatology, Royal Victoria Infirmary, Newcastle upon Tyne NHS Hospitals National Health Service Foundation Trust, Newcastle upon Tyne, United Kingdom.; 19 Institute of Translational and Clinical Medicine, Faculty of Medical Sciences, Framlington Place, Newcastle University, Newcastle upon Tyne, United Kingdom.; 20 Health Data Research UK, London, United Kingdom.

**Keywords:** Genetics, Therapeutics, Adaptive immunity, Drug therapy, Molecular genetics

## Abstract

Targeted biologic therapies can elicit an undesirable host immune response characterized by the development of antidrug antibodies (ADA), an important cause of treatment failure. The most widely used biologic across immune-mediated diseases is adalimumab, a tumor necrosis factor inhibitor. This study aimed to identify genetic variants that contribute to the development of ADA against adalimumab, thereby influencing treatment failure. In patients with psoriasis on their first course of adalimumab, in whom serum ADA had been evaluated 6–36 months after starting treatment, we observed a genome-wide association with ADA against adalimumab within the major histocompatibility complex (MHC). The association signal mapped to the presence of tryptophan at position 9 and lysine at position 71 of the HLA-DR peptide-binding groove, with both residues conferring protection against ADA. Underscoring their clinical relevance, these residues were also protective against treatment failure. Our findings highlight antigenic peptide presentation via MHC class II as a critical mechanism in the development of ADA against biologic therapies and downstream treatment response.

## Graphical abstract

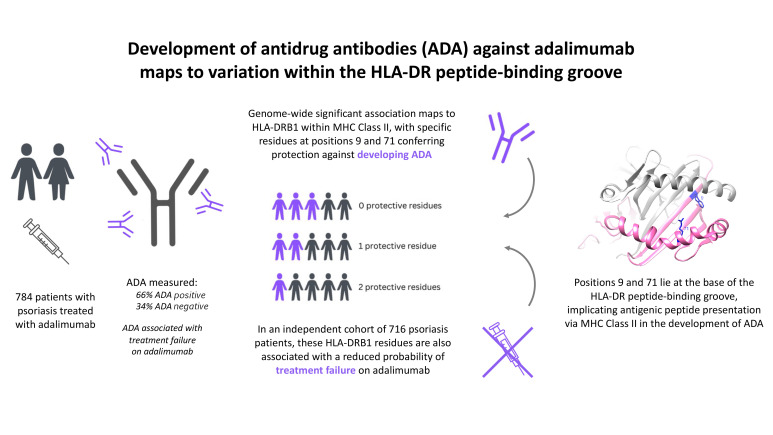

## Introduction

Monoclonal antibodies comprise the largest family of biologic therapies and have transformed the treatment of immune-mediated inflammatory diseases (IMIDs). However, serial administration of recombinant protein drugs may induce an undesirable immune response characterized by development of antidrug antibodies (ADA) (1, 2). These ADA neutralize the drug and/or cause increased clearance of ADA-drug complexes, leading to suboptimal drug exposure and treatment failure. ADA may also increase risk of toxicity due to loss of drug targeting or formation of highly immunogenic complexes (3).

Estimates of ADA prevalence vary widely depending on the drug, disease, and ADA assay technique, but up to 65% of patients with IMID reportedly develop ADA in response to biologics inhibiting the pro-inflammatory cytokine tumor necrosis factor (TNF) (1, 4–6). Here the relevance of ADA is well established and increasingly monitored in the clinical setting; a meta-analysis reported that ADA against infliximab or adalimumab reduced drug response rate by 68%, an effect attenuated by concomitant immunosuppression with methotrexate (7).

Although several drug-related factors influence drug immunogenicity, not all individuals develop significant immune responses to a given biologic. This implies the existence of host-specific factors that influence variability in the antidrug response in vivo. Major histocompatibility complex (MHC) class II genes have been hypothesized to be centrally involved in the development of ADA, fueling a series of candidate gene studies reporting associations with ADA against TNF inhibitors (8–10). More recently, systematic genome-wide analyses of ADA against TNF inhibitors have been performed in a range of clinical contexts (11, 12); however, the only reported association with ADA to surpass the multiple-testing threshold for genome-wide significance is at HLA-DQA1*05 in individuals with inflammatory bowel disease (IBD) taking either infliximab or adalimumab (12).

Psoriasis provides an optimal clinical scenario to study the genetic basis of ADA development, since it is one of the most common indications for biologic use, with adalimumab a first-line treatment. Critically, biologics for psoriasis are generally used as monotherapy, whereas patients with IBD and rheumatoid arthritis (RA) receiving the same biologic therapies are often coprescribed immunosuppressants, which are known to reduce the development of ADA.

The current investigation represents the largest genetic study of ADA against adalimumab and to our knowledge the first genetic investigation of ADA against any biologic in psoriasis. In a real-world data set, we examine the role of genetic variation across the genome and implicate antigenic peptide presentation via MHC class II as a key genetic driver of ADA development. We further demonstrate that the genetic variation associated with ADA development is also associated with clinical outcome, since individuals harboring alleles protective against ADA were also less likely to terminate adalimumab due to ineffectiveness.

## Results

### GWAS of adalimumab ADA in individuals with psoriasis sampled 6–36 months after treatment initiation.

**Figure 1:**
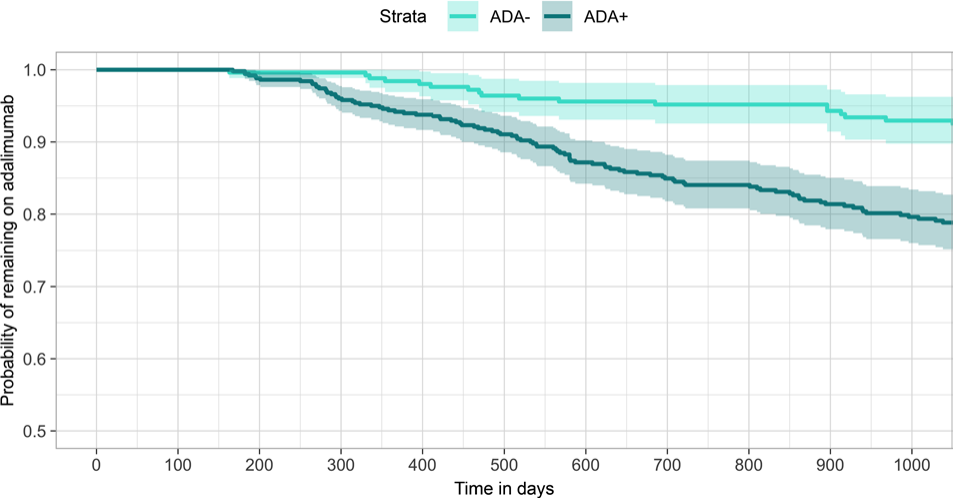
Time to termination of adalimumab treatment due to ineffectiveness, stratified by ADA status (discovery cohort: individuals sampled 6–36 months after treatment initiation, *n* = 784). Light turquoise: ADA negative (undetectable). Dark turquoise: ADA positive (detectable). Kaplan-Meier survival analysis was used.

Previous studies of the immune response to adalimumab indicate that the majority of ADA-positive cases are detectable by 6 months after treatment initiation (13, 14). To maximize detection of ADA-positive individuals for our discovery analysis, we performed ADA detection using a drug-tolerant assay in a cohort of 784 individuals for whom a serum sample had been obtained 6–36 months after starting adalimumab (mean 418 days, range 183–1,095 days). ADA was detected in 521 (66%) individuals, whereas 263 (34%) had no detectable ADA (see Supplemental Figure 1 and Supplemental Table 1; supplemental material available online with this article; https://doi.org/10.1172/jci.insight.156643DS1). Consistent with previous investigations, those testing positive for ADA were also more likely to terminate treatment due to ineffectiveness (HR 2.31, 95% CI 1.59–3.36, *P* = 9 × 10^–6^; Figure 1).

**Figure 2:**
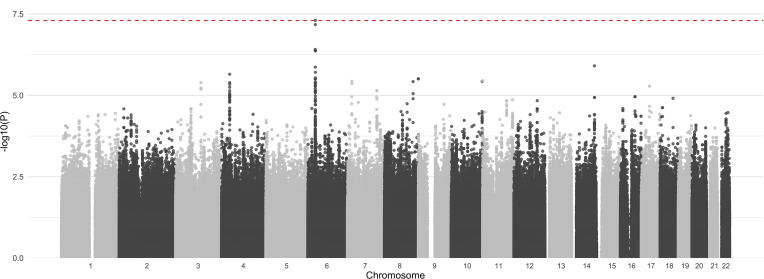
Evidence for association with ADA development (–log_10_ *P* value) from a genome-wide analysis of 10,917,604 genetic variants (discovery cohort: individuals sampled 6–36 months after treatment initiation, *n* = 784). Chromosomes shown in alternating shading; red line indicates the threshold for genome-wide significance (*P* = 5 × 10^–8^). Logistic regression was used.

Genome-wide genotyping was performed in 784 individuals of European ancestry, for whom ADA status had been determined 6–36 months after starting adalimumab. Following quality control and genome-wide imputation of common variants with the Haplotype Reference Consortium panel (15, 16), we tested 10,917,604 autosomal genetic variants for association with ADA status (Figure 2 and Supplemental Figure 2). We observed evidence of association with a series of genetic variants in high linkage disequilibrium (LD) within the MHC region at 6p21.32. The genetic variant with the strongest evidence of association (rs9268628, OR 3.44, 95% CI 2.21–5.36, *P* = 4.93 × 10^–8^) is a single nucleotide substitution located within the cluster of genes encoding MHC class II antigen-presenting molecules.

### Fine-mapping of the MHC association signal to HLA-DRB1 residues 9 and 71.

To fine-map the causal genetic variation underlying the observed association within the MHC, we imputed genetic variation within the protein-coding regions of genes encoding MHC class II antigen-presenting molecules (HLA-DPQ1, -DPB1, -DQA1, -DQB1, -DRB1), inferring the resulting genotypes of variable amino acid residues and classical alleles at 2- and 4-digit resolution. The resulting genotypes of classical alleles and combinations of amino acid residues at specific positions were tested for association with ADA (see Supplemental Table 5). The strongest evidence of association was observed at position 9 of HLA-DRB1, where the presence of a tryptophan residue conferred protection against ADA (OR 0.57, 95% CI 0.46–0.71, *P* = 3.09 × 10^–7^), and at position 11, where the presence of either a serine, valine, or aspartic acid residue was associated with risk of developing ADA (OR 1.74, 95% CI 1.40–2.16, *P* = 3.09 × 10^–7^). These specific allelic combinations at positions 9 and 11 were in perfect LD and anticorrelated. No single classical allele of any of the 5 class II molecules had stronger evidence of association with ADA, although we noted HLA-DRB1*11 to be the classical allele with the strongest evidence of association with ADA (OR 3.38, 95% CI 1.70–6.71, *P* = 6 × 10^–4^, see Supplemental Table 6). No evidence of association was observed for HLA-DQA1*05 (OR 1.29, 95% CI 0.98–1.71, *P* = 0.07), which has previously been reported to be associated with ADA against TNF inhibitors in a population with IBD (12).

**Figure 3:**
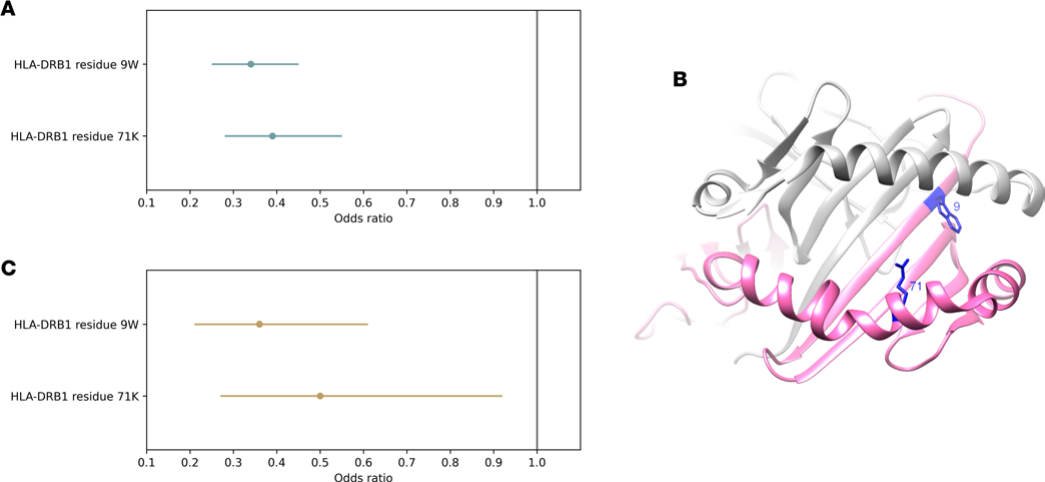
Association of ADA development with HLA-DRB1 residues 9 and 71 within the peptide-binding groove. (**A**) Estimated odds ratios for the presence of a tryptophan residue at position 9 (9W) and a lysine residue at position 71 (71K) of HLA-DRB1 on risk of ADA development in the discovery cohort (individuals sampled 6–36 months after treatment initiation, *n* = 784). Effect sizes and 95% confidence intervals estimated jointly for both variants in a multiple regression model. (**B**) Three-dimensional ribbon model of the HLA-DR protein. Structure based on Protein Data Bank entry 3pdo, with a direct view of the peptide-binding groove. HLA-DRB is shown in pink; HLA-DRA is shown in gray. The 2 key amino acid positions identified by the association analyses are shown with their side chains and highlighted in blue. (**C**) Estimated odds ratios for the presence of a tryptophan residue at position 9 (9W) and a lysine residue at position 71 (71K) of HLA-DRB1 on risk of ADA development in the replication cohort (individuals sampled within 6 months of treatment initiation, *n* = 232). Effect sizes and 95% confidence intervals estimated jointly for both variants in a multiple regression model.

We conducted an association analysis conditioned on the presence of a tryptophan residue at position 9; this revealed a secondary association signal at position 71, where the presence of a lysine residue also conferred protection against ADA (joint model; position 9 tryptophan: OR 0.34, 95% CI 0.25–0.45, *P* = 1.76 × 10^–12^; position 71 lysine: OR 0.39, 95% CI 0.28–0.55, *P* = 5.77 × 10^–8^; Figure 3A). The original genome-wide significant association observed with rs9268626 was strongly attenuated when tryptophan at position 9 and lysine at position 71 were included in the regression model (rs9268626 conditional OR 2.0, 95% CI 1.23–3.28, *P* = 0.0048). The effects of tryptophan at HLA-DRB1 position 9 and lysine at position 71 were robust to potential confounders, including immunosuppressant cotherapy, threshold of ADA detection, genotype of the psoriasis susceptibility allele HLA-C*06:02, sample timing, comorbidities (including psoriatic arthritis and diabetes), age, age of psoriasis onset, sex, and baseline disease severity (see Supplemental Table 6).

The association analysis indicated that the combination of alleles at positions 9 and 71 of HLA-DRB1 represented the most parsimonious explanation for the original observed association with ADA. Positions 9 and 71 are both located at the base of the HLA-DR peptide-binding groove (Figure 3B) and have been shown to influence the binding specificity of their respective major binding pockets (P9 pocket for position 9, P4 pocket for position 71) (17, 18). The amino acid residues conferring protection against ADA at positions 9 and 71 (tryptophan and lysine, respectively) have not been observed together within a single reported classical HLA-DRB1 allele to our knowledge, and imputation of these alleles in the discovery cohort identified no individuals carrying more than 2 protective alleles across these 2 positions within HLA-DRB1 (i.e., on each haplotype, individuals could have a tryptophan at position 9 or a lysine at position 71, but not both). The effect of the protective alleles was additive, with each allele conferring approximately 2.8-fold protection against ADA development (OR 0.36, 95% CI 0.27–0.48, *P* = 3 × 10^–12^). Consistent with these observations, classical HLA-DRB1 alleles harboring either a tryptophan at position 9, or a lysine at position 71, were enriched for protective effects against ADA development (see Supplemental Table 7). Conversely, the classical allele with the strongest positive association with ADA risk, HLA-DRB1*11, did not harbor either a tryptophan at position 9 or a lysine at position 71.

### Replication of effect of HLA-DRB1 residues 9 and 71 on development of ADA in individuals with psoriasis sampled within 6 months of treatment initiation.

In an independent set of 232 individuals, we sought to replicate the observed protective effects of HLA-DRB1 residues 9 and 71 on ADA status using serum samples obtained within 6 months of starting adalimumab (mean 100 days, range 2–182 days, see Supplemental Table 2). ADA was detected in 150 (65%) individuals, whereas 82 (35%) individuals had no detectable ADA according to the drug-tolerant assay. We noted that the proportion of individuals testing positive for ADA was low in those sampled within the first 2 months (34%) but increased to levels (72%) similar to those observed between months 6 and 18 in the discovery cohort (see Supplemental Figure 1). Within this replication cohort, association testing of the joint effect of HLA-DRB1 positions 9 and 71 on ADA detected within the first 6 months revealed a direction and magnitude of effect consistent with that observed in the discovery cohort (position 9: OR 0.36, 95% CI 0.21–0.61, *P* = 2.72 × 10^–5^; position 71: OR 0.50, 95% CI 0.27–0.92, *P* = 0.027; Figure 3C).

### Association of HLA-DRB1 residues 9 and 71 with adalimumab treatment failure.

**Figure 4:**
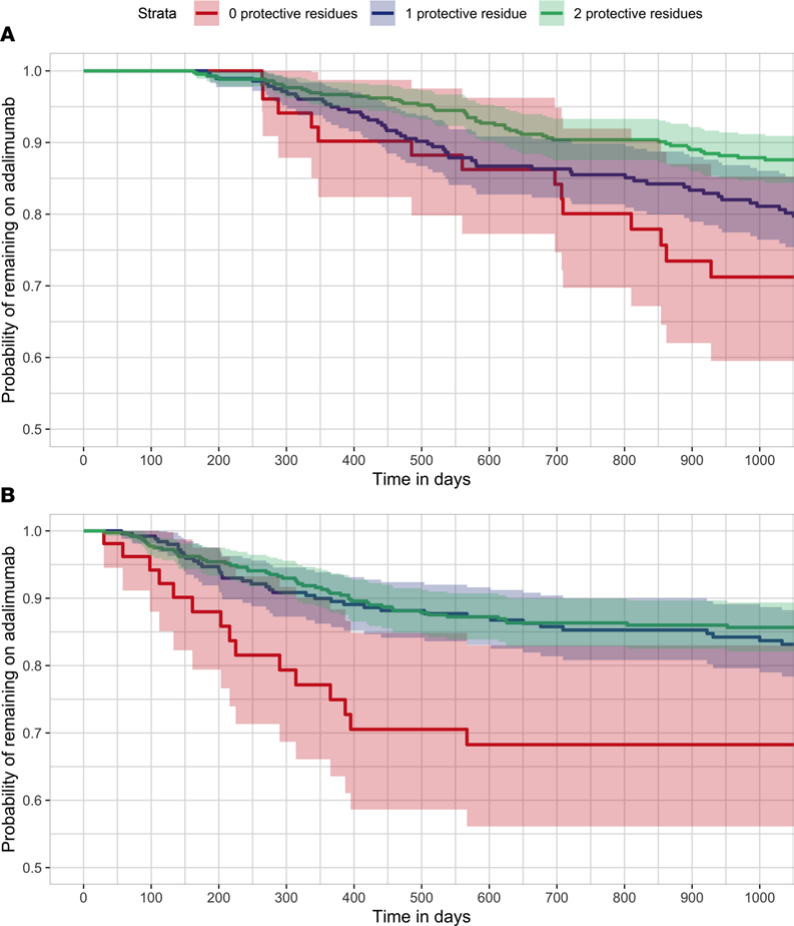
Time to termination of adalimumab treatment due to ineffectiveness, stratified by the number of HLA-DRB1 protective residues present. (**A**) Within the discovery cohort (individuals sampled 6–36 months after treatment initiation, *n* = 784). (**B**) Within the adalimumab treatment duration cohort (individuals for whom clinical data but no ADA status was available, *n* = 716). Kaplan-Meier survival analysis was used. Red: 0 protective residues. Blue: 1 protective residue. Green: 2 protective residues.

Drug immunogenicity has previously been associated with both nonresponse and loss of response to adalimumab treatment (1, 7). Indeed, in our discovery cohort, we observed that individuals with detectable ADA were significantly more likely to terminate treatment due to ineffectiveness (HR 2.31, 95% CI 1.59–3.36, *P* = 9 × 10^–6^; Figure 1). Consistent with this association, we observed that the ADA-protective residues within HLA-DRB1 were also protective against treatment failure, since individuals with ADA-protective residues within HLA-DRB1 were significantly less likely to terminate treatment due to ineffectiveness (HR 0.77, 95% CI 0.61–0.97, *P* = 0.03; Figure 4A).

We next sought to evaluate this association in an independent set of 716 individuals on adalimumab for psoriasis, for whom ADA status was not available (see Supplemental Table 3). Again, the observed effect of the HLA-DRB1 residues at positions 9 and 71 on treatment failure was consistent with their effect on ADA development; ADA-protective residues were associated with reduced probability of terminating the adalimumab treatment due to ineffectiveness (HR 0.73, 95% CI 0.57–0.94, *P* = 0.013; Figure 4B). The effect estimates of the 2 protective residues on clinical outcome were not substantially influenced by adjusting for age, sex, and weight (see Supplemental Figure 3). Individuals harboring 1 ADA-protective residue had 0.90 (95% CI 0.86–0.94) probability of remaining on adalimumab for 1 year and 0.83 (95% CI 0.78–0.88) probability of remaining on adalimumab for 3 years, with very similar effects in individuals harboring 2 protective residues (0.91 [95% CI 0.88–0.94] probability of remaining on adalimumab to 1 year; 0.85 [95% CI 0.82–0.89] probability of remaining on adalimumab for 3 years). In contrast, individuals harboring no protective residues had 0.75 probability (95% CI 0.64–0.88) of remaining on adalimumab for 1 year and 0.68 (95% CI 0.56–0.83) probability of remaining on adalimumab for 3 years.

## Discussion

Drug immunogenicity is a substantial clinical concern, as individuals who develop ADA are at greater risk of treatment failure (7, 14, 19, 20). In our cohort, individuals with ADA were about 2.5 times more likely to terminate their adalimumab treatment regime due to ineffectiveness, compared with those with no detectable ADA. Despite advances in the drug immunogenicity field, clinical ADA monitoring is challenging due to lack of standardization across ADA assays and no accepted thresholds for clinically relevant titers influencing drug level or treatment response. Therefore, the identification of an easily assayed genetic biomarker associated with ADA development and treatment outcome ahead of therapeutic intervention may hold potential for clinical and economic benefit.

In the largest genetic study of ADA against adalimumab to date, we identify genetic variation within the MHC class II region on chromosome 6 as the primary genetic determinant of ADA development in patients with psoriasis. Fine-mapping of the causal genetic variation to a combination of residues in the peptide-binding groove of HLA-DR highlighted the importance of antigen presentation by this MHC class II molecule in the adaptive immune response to biologic therapies. Critically, we observed that the same genetic variation was also associated with treatment termination due to ineffectiveness.

Our results implicate positions 9 and 71 of HLA-DRB1 in susceptibility to the development of ADA against adalimumab, with the presence of a tryptophan residue at position 9 and a lysine residue at position 71 conferring protection against ADA. Due to the perfect co-occurrence of tryptophan at position 9 with the absence of serine, valine, or aspartic acid residues at position 11 within HLA-DRB1 sequences, we cannot exclude the possibility that the causal residues within HLA-DRB1 include those at position 11, instead of or in combination with those at positions 9 and 71. The associated HLA-DRB1 residues are each located at the base of the peptide-binding groove and within major binding pockets: positions 9 (P9 pocket) and 11 (P6 pocket) are located on the β-sheet floor, with their side chains oriented into the groove; position 71 (P4 pocket) is separated by a single turn along the α helix, with its side chains spatially close to those of position 11 (21). Antigen-presenting MHC class II proteins have been implicated in the development of ADA against several biologic therapies, with HLA-DRB1 reported to be associated with ADA in several clinical settings (8–10, 22–26). Although reported associations often do not meet the levels of statistical rigor demanded by contemporary genetic studies, a notable exception is the reported association of HLA-DQA1*05 with the development of ADA against infliximab and adalimumab in individuals with IBD (11). However, this observation is not without controversy, with post hoc treatment-specific analysis of the same data indicating that HLA-DRB1*11:01 confers specific risk for the development of ADA against adalimumab, with no evidence of association with HLA-DQA1*05:01 for this drug (27). This failure to observe an HLA-DQA1*05:01 association with adalimumab ADA has been attributed to a lack of statistical power in the IBD adalimumab cohort (28). In the current study, we note the effect of HLA-DRB1*11 on adalimumab ADA risk, together with a lack of evidence for association of DQA1*05.

The specific HLA-DRB1 amino acid positions highlighted by our analysis are well established in numerous disease settings where adaptive immunity plays a critical role. Although there have been no reports of association with psoriasis susceptibility, which is primarily driven by the MHC class I allele HLA-C*06:02 (29), in RA a substantial proportion of disease risk is explained by specific residues at positions 11, 71, and 74 of HLA-DRB1 (30). Positions 11 and 71 of HLA-DRB1 have also been postulated to influence risk of IBD (17, 31), type 1 diabetes (32, 33), MS (34), sarcoidosis (35), and visceral leishmaniasis (36). However, to the best of our knowledge, there have been no reports of HLA-DRB1 disease associations that resolve to the same combination of alleles at positions 9, 11, and 71 that we describe here.

The use of a drug-tolerant assay is a critical aspect of the current study design, due to the predicted impact of variability in sample timing, with respect to treatment administration, on serum drug levels. The proportion of individuals testing positive for ADA against adalimumab was higher than previously reported (5, 6, 14); this is consistent with the increased sensitivity of the drug-tolerant ADA assay (4), and may also reflect lower levels of cotherapy with immunosuppressants such as methotrexate in psoriasis compared with other IMIDs. Given the challenges of accurate ADA quantification, we modeled ADA as a binary outcome using the threshold for detection of ADA from the assay manufacturer; sensitivity analysis using a higher assay threshold did not impact the primary finding (4). Due to the constraints of the sampling scheme, we did not attempt to model time to detection of ADA using survival analysis. This analysis approach holds potential for bias, namely that assessment of ADA at a single time point may mean that some individuals classified as negative for ADA will go on to develop ADA, reducing the statistical power to detect association. However, sensitivity analysis indicated that sample timing did not influence the observed association of HLA-DRB1 residues on ADA risk. The study design did not evaluate ADA status at baseline, prior to starting adalimumab, although all patients were adalimumab naive and would not be expected to be ADA positive prior to drug exposure.

We acknowledge the limitations of the study cohort, which includes only individuals of European ancestry with severe psoriasis in the United Kingdom. The inclusion of individuals consenting to different levels of bioresourcing may have introduced selection bias, although baseline demographics are broadly comparable across the individual cohorts. Replication and further investigation in cohorts with larger sample sizes and those encompassing different population ancestries may be informative to disentangle the individual effects of amino acid residues at positions 9 and 11.

Adalimumab remains a first-line agent across multiple IMIDs; its recent off-patent status in Europe, its impending patent expiration in the United States, and the growing development of adalimumab biosimilars are converging on high availability of this drug, with a corresponding drop in cost. Even small gains in optimizing use of adalimumab and its emerging biosimilars could translate into clinical and economic benefit. Although further validation is required, pretreatment HLA-DRB1 genotyping to stratify patients according to risk of developing ADA may have clinical utility in guiding treatment selection. In patients lacking ADA-protective HLA alleles, less immunogenic but higher cost drugs may be considered as alternatives to adalimumab (1), with a lower threshold to start concomitant immunosuppression. Conversely, in individuals with ADA-protective HLA alleles, adalimumab could be used first-line. Finally, we note that our findings appeared to be independent of HLA-C*06:02 status, which not only makes the strongest genetic contribution to psoriasis susceptibility (37) but critically also predicts response to both adalimumab and the IL-12/23 inhibitor ustekinumab (38). Understanding the mechanisms through which the observed association impacts ADA development is an important next step. One avenue for investigation is identification of the peptides, whether self-peptides, adalimumab fragments, or other exogenous peptides, that are differentially bound by alleles harboring tryptophan at position 9 and lysine at position 71. These may provide insight into whether the observed association with HLA-DRB1 and ADA against adalimumab also predicts the development of ADA against adalimumab in other disease settings and the development of ADA against other systemic medications for the treatment of psoriasis.

The robust identification of genetic drivers of ADA development and ineffectiveness of treatment should now motivate development of integrated treatment prediction models across genetic and clinical biomarkers, to optimize treatment selection and maximize patient benefit in psoriasis and other IMIDs.

## Methods

### ADA discovery and replication cohort selection.

The discovery cohort comprised 784 individuals in the BSTOP cohort for whom genotyping data were available and a serum sample was obtained 6–36 months after starting their first course of adalimumab (see Supplemental Table 1). The replication cohort comprised 232 individuals who did not meet the sampling criteria for the discovery cohort but for whom a serum sample was obtained within the first 6 months of starting their first course of adalimumab (see Supplemental Table 2).

### Adalimumab treatment duration cohort selection.

Evaluation of genetic association with treatment failure was evaluated in 716 individuals for whom a serum sample had not been collected for detection of ADA but for whom genotyping data, treatment dates, and clinical outcome data were available (see Supplemental Table 3).

### Treatment definitions.

All patients were on the standard adalimumab dose of 40 mg every 2 weeks at the time of ADA sampling. Adalimumab therapy was considered ongoing where treatment episodes were separated by fewer than 90 days (38). Immunosuppressant cotherapy while on adalimumab was defined as receiving an immunosuppressant drug for psoriasis during the first course of adalimumab and before the date of the serum sample. Duration of treatment was measured in days since the first dose of adalimumab.

### ADA detection.

Venous blood samples were collected during routine clinic visits; serum was extracted following centrifugation at 2,000*g* for 10 minutes and stored at –80°C prior to adalimumab ADA detection using a drug-tolerant assay (acid dissociation radioimmunoassay, ARIA). A threshold of ≥30 arbitrary units/mL (4) was used to define detectable ADA.

### Genotyping and imputation.

Genomic DNA was extracted from whole blood, and genotyping was performed using the Illumina HumanOmniExpressExome-8 v1.2 and v1.3 and v1.6 BeadChips (Illumina). Genotype calling was performed using Illumina’s GenomeStudio Data Analysis software. Quality control was performed using established protocols (39–41). Genome-wide SNP imputation was undertaken using the Haplotype Reference Consortium (version r1.1) reference panel (15) on the Michigan Imputation Server (16); imputation of 221 classical alleles and 1,052 amino acid residues was undertaken using SNP2HLA (version 1.0.3) software and the Type I Diabetes Genetics Consortium reference panel (42). Analysis was restricted to variants with high imputation quality (*r*^2^ > 0.9).

### Statistics.

Evaluation of the association of genetic variants (37 two-digit classical alleles, 56 four-digit classical alleles, and 516 amino acid residues) and ADA status was performed using a logistic regression model in PLINK and R (40). The first 5 principal components were included in the model to control for potential population stratification.

A Cox proportional hazard model was used to test the association between the likelihood of terminating adalimumab due to ineffectiveness and either ADA status or amino acid residues at positions 9 and 71 of HLA-DRB1. Individuals terminating adalimumab for reasons other than ineffectiveness were censored at the adalimumab end date; those without a stop date (due to continuing on adalimumab at data set cutoff or loss to follow-up) were censored at the last visit date. The assumption of proportionality was tested using Schoenfeld residuals. The Kaplan-Meier method was used to estimate the probability of remaining on the drug for 1, 2, or 3 years without terminating the drug due to ineffectiveness.

A series of sensitivity analyses were performed to evaluate the impact of immunosuppressant cotherapy, threshold of detection in the ADA assay, HLA-C*06:02 genotype, sample timing, presence of comorbidities (including psoriatic arthritis and diabetes), age, age of psoriasis onset, sex, and baseline psoriasis severity (see Supplemental Table 4).

### Study approval.

BSTOP is a UK prospective observational multicenter (*n* = 87) cohort study, approved by the South East London REC 2 Ethics Committee, London, United Kingdom (11/H0802/7), conducted in the spirit of the 1996 International Conference on Harmonisation in Good Clinical Practice and in accordance with the 2008 Declaration of Helsinki. Written informed consent was obtained from all participants prior to enrollment to the BSTOP bioresource (43), including consent for access to clinical data stored in the British Association of Dermatologists Biologic and Immunomodulators Register (BADBIR; approved by the NHS Research Ethics Committee North West England, Manchester, United Kingdom; 07/MRE08/9) (44). Enrollment criteria are reported in detail elsewhere (44) and include age more than 16 years, dermatologist’s diagnosis of psoriasis, and treatment with conventional systemic or biologic therapy.

## Author contributions

TT, CS, and MAS were responsible for the study concept and design. TT performed all analyses and wrote the manuscript. TR, FCL, and ADV contributed to study design and provided expertise on antidrug antibody biology and assays. MHA, IAB, DB, TD, MD, and FM were responsible for managing BSTOP data and samples. ND was responsible for the BSTOP genotyping data set (quality control, imputation, and data management), contributed to study design, and provided scientific input on data analyses and interpretation. JB provided scientific input on study design and data interpretation. TT, JS, TR, FCL, ADV, MHA, IAB, DB, TD, MD, FM, AR, AC, S McBride, KM, GP, HR, RR, KS, AS, ADB, CEMG, NJR, RBW, S Mahil, JB, ND, CS, and MAS provided critical revision of the manuscript for intellectual content.

## Supplementary Material

Supplemental data

## Version 1 02/22/2023

Electronic publication
