# Supplementary material for: Development of antidrug antibodies against adalimumab maps to variation within the HLA-DR peptide-binding groove
Source: JCI Insight. 2023 Feb 22;8(4):e156643. doi: 10.1172/jci.insight.156643 (PMC9977494; doi:10.1172/jci.insight.156643)
Supplement: Supplemental data [file jciinsight-8-156643-s063.pdf]

Supplemental Material

Acknowledgements

Members of the **Biomarkers of Systemic Outcomes in Psoriasis (*BSTOP*) Study Group** are as follows:

PI Name	Trust
Aamir Butt	North Lincolnshire and Goole NHS Trust
Ahmed Al-Rusan	Hywel Dda Health Board
Alexa Shipman	Portsmouth Hospital NHS trust
Alexandra Bonsall<br>Fiona Meredith	NHS Grampian
Alison Godwin<br>Diane Williamson	Betsi Cadwala University Health Board
Alison Layton	Harrogate and District NHS Foundation Trust
Andrew Wright	Bradford Teaching Hospitals NHS Foundation Trust
Anthony Bewley	Whipps Cross University Hospital NHS Trust
Aruni Ranasinghe	West Suffolk NHS Foundation Trust
Bernadette DeSilva	Luton & Dunstable University Hospital NHS Foundation Trust
Calum Lyon	York teaching Hospital NHS Foundation Trust
Caoimhe Fahy<br>Chris Lovell	Royal United Hospitals Bath NHS Foundation Trust
Caroline Angit	United Lincolnshire Hospitals NHS Trust
Caroline Owen	East Lancashire Hospitals NHS Trust
Charles Archer<br>Caroline Higgins	Royal Berkshire NHS Foundation Trust
Claudia DeGiovanni	Brighton and Sussex University Hospitals NHS Trust
Emily Davies	Gloucestershire Hospitals NHS Foundation Trust
Eva Domanne	Countess of Chester Hospital NHS Foundation Trust
Fiona Antony	Frimley Park Hospital NHS Foundation Trust
Fiona Hammonds	Royal Cornwall Hospitals NHS Trust
Gay Perera	Westminster Hospital NHS Foundation Trust
Helen Lewis	University Hospitals Birmingham NHS Foundation Trust
Helen Ramsay	Sheffield Teaching Hospitals NHS Foundation Trust
Ingrid Salvary	James Paget University Hospitals NHS Foundation Trust
Jon Goulding	Heart of England NHS Foundation Trust
Kathy Sands	East Kent Hospitals University NHS foundation Trust
Kathy Taghipour	The Whittington Hospitals NHS Foundation Trust
Katya Burova	Bedford Hospital NHS Foundation Trust
Kavitha Sundararaj<br>Anna Chapman	South London Healthcare NHS Trust
Kay Baxter	Barnsley Hospital NHS Foundation Trust
Kevin McKenna	Belfast Health and Social Care Trust
Leila Ferguson<br>Ruth Lamb	St George's University Hospitals NHS Foundation Trust
Mahmud Ali	Western Sussex Hospitals NHS Foundation Trust
Manju Kalavala	Cardiff and Vale University Health Board
Maria-Angeliki Gkini	Whipps Cross University Hospital NHS Trust
Martin Wade	Barking, Havering and Redbridge University Hospitals NHS Trust

Michelle Thomson<br>Raakhee Ramesh	Sandwell & West Birmingham Hospitals NHS Trust
Mihaela Costache	South Devon Healthcare NHS Foundation Trust
Mohsen Khorshid	Basildon and Thurrock University Hospitals NHS Foundation Trust
Nabil Ponnambath	Aneurin Bevan Health Board
Nadia Aldoori	Mid Yorkshire Hospitals NHS Trust
Nick Levell	Norfolk and Norwich University Hospitals NHS Foundation Trust
Paul Norris<br>Shaheen Haque	Cambridge University Hospitals NHS Foundation Trust
Periasamy Balasubramaniam	Betsi Cadwala University Health Board
Rachel Wachsmuth	Yeovil District Hospital NHS Foundation Trust
Rada Rotarescu	University Hospital of North Midlands NHS Trust
Rebecca Rose	Mid Yorkshire Hospitals NHS Trust
Richard Parslew	Liverpool University Hospitals NHS Foundation Trust
Roberto Verdolini	Princess Alexan a Hospitals NHS Trust
Ruth Murphy	Nottingham University Hospitals NHS Trust
Sally McCormack	NHS Fife
Sandeep Cliff	Surrey and Sussex Healthcare Trust
Sandy McBride	Royal Free London NHS Foundation Trust
Sarah Walsh	King's College Hospital NHS Foundation Trust
Serap Mellor	Salisbury NHS Foundation Trust
Sharmela Darne	South Tees Hospitals NHS Foundation Trust and North Tees and Hartlepool Hospitals NHS Foundation Trust
Shernaz Walton	Hull and East Yorkshire Hospitals NHS Trust
Shyamal Wahie	County Durham and Darlington NHS Foundation Trust
Simina Stefanescu	Queen Elizabeth Hospital King's Lynn NHS Foundation Trust
Suzanna August	University Hospitals Dorset NHS Foundation Trust
Tapati Sinha	Southport and Ormskirk Hospital NHS Trust
Teresa Joseph	Sherwood Forest Hospitals NHS Foundation Trust
Trupti Desai	Doncaster and Bassetlaw Hospitals NHS Foundation Trust
Urvi Popli	Betsi Cadwala University Health Board
Victoria Brown	West Hertfordshire Hospitals NHS Trust
Victoria Diba<br>James Powell	Wye Valley NHS Trust
Areti Makrygeorgou	NHS Greater Glasgow and Clyde
Harvey Dymond	Weston Area Health NHS Trust
Joanne Topliffe	Mid Essex Hospital Services NHS Trust
Louise Wilcox	Burton Hospitals NHS Foundation Trust
Mel Caswell	Blackpool Teaching Hospitals NHS Foundation Trust
Michelle Taylor	The Dudley Group NHS Foundation Trust
Sarah Johnson	Mid Staffordshire NHS Foundation Trust and Royal Wolverhampton NHS Trust
Nicole Lawson	Mid Cheshire Hospitals NHS Foundation Trust

Alex Anstey	Betsi Cadwala University Health Board
Catherine Smith	Guy's and St. Thomas' NHS Foundation Trust
Nick Reynolds	Newcastle Upon Tyne Hospitals NHS Foundation Trust
Richard Warren	Salford Royal NHS Foundation Trust
Saskia Reeken	Kingston NHS Foundation Trust
Sue Hood	The Ipswich Hospital NHS Trust
Tara Lees<br>Susanne Farwer	Rotherham NHS Foundation Trust

Members of the **British Association of Dermatologists Biologic and Immunomodulators Register (*BADBIR*) Study Group** include Jonathan Barker, Marilyn Benham, David Burden, Ian Evans, Christopher Griffiths, Sagair Hussain, Brian Kirby, Linda Lawson, Kayleigh Mason, PhD, Kathleen McElhone, Ruth Murphy, Anthony Ormerod, Caroline Owen, Nick Reynolds, Catherine Smith, and Richard Warren.

Members of the **Psoriasis Stratification to Optimise Relevant Therapy (*PSORT*) Consortium** include Jonathan Barker, Michael R. Barnes, A. David Burden, Paola di Meglio, Richard Emsley, An ea Evans, Christopher E.M. Griffiths, Katherine Payne, Nick J. Reynolds, Catherine Smith, Deborah Stocken, and Richard B. Warren.

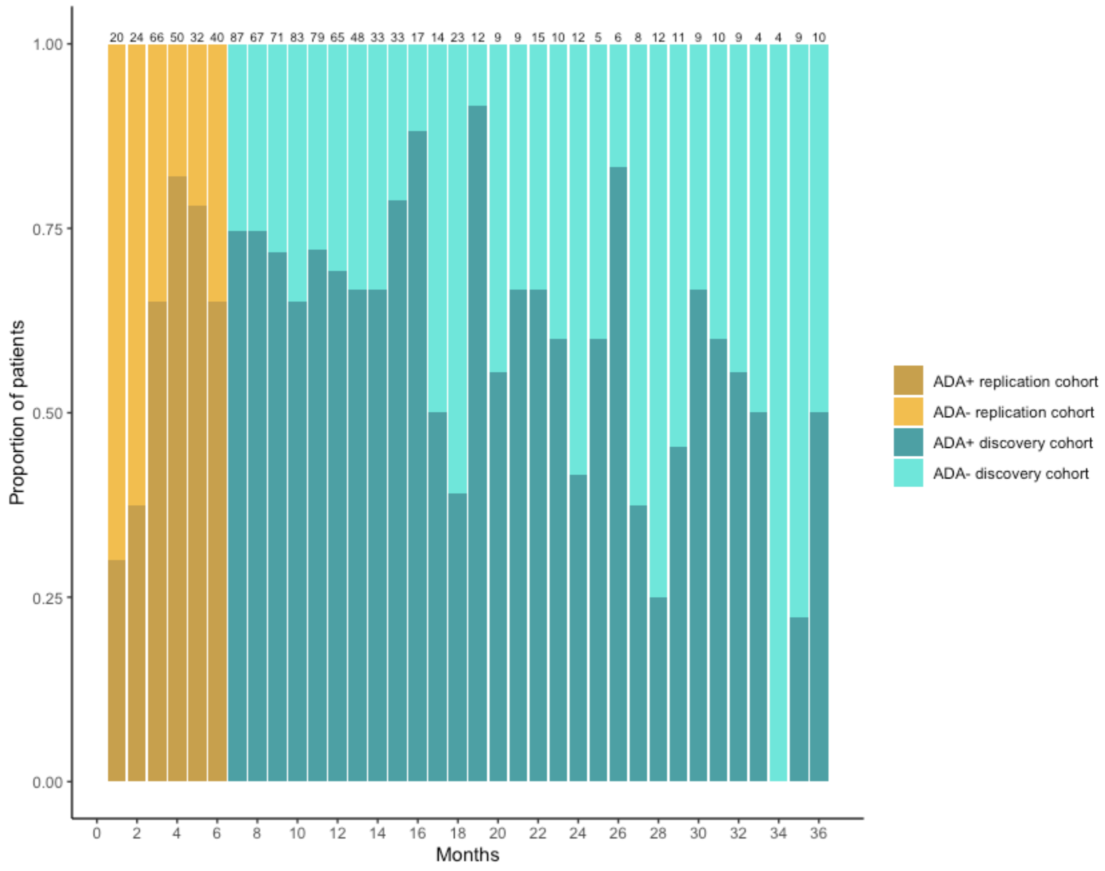

Figure E1. ADA prevalence over time. Proportion of patients that are ADA+ (dark coloured bars); ADA- (light coloured bars) by month after treatment initiation. Yellow denotes the replication cohort (individuals sampled within 6 months of treatment initiation, n=232). Turquoise denotes the discovery cohort (individuals sampled 6-36 months after treatment initiation, n=784).

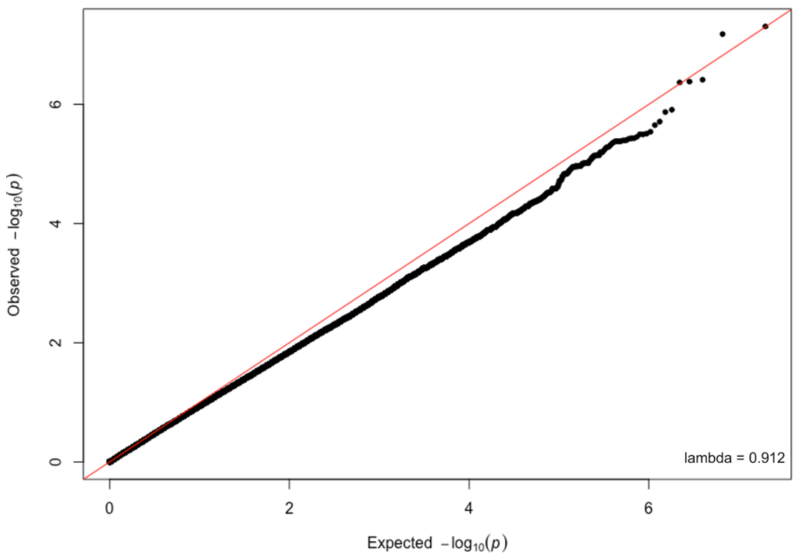

Figure E2. Quantile-quantile plot of ADA association p values (discovery cohort: individuals sampled 6-36 months after treatment initiation, n=784).

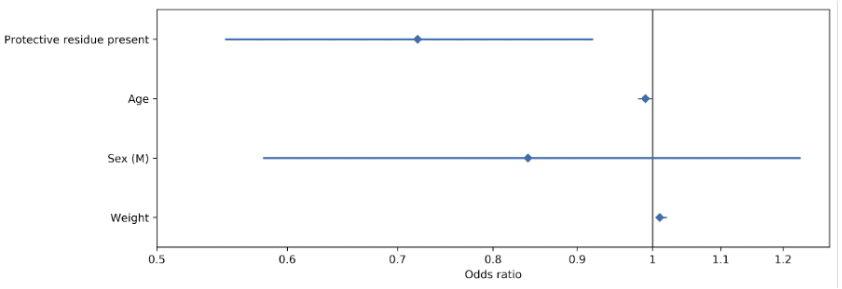

Figure E3. Effect sizes for each covariate in Cox regression model for termination of adalimumab due to ineffectiveness (adalimumab treatment duration cohort: individuals with clinical data but no ADA status available, n=716).

Table E1. Baseline characteristics of 784 individuals sampled 6-36 months after starting first course of adalimumab (discovery cohort). ADA+: detectable ADA, ADA-: undetectable ADA.

	All	ADA+	ADA-	Missing data
Number	784	521 (66%)	263 (34%)	-
Age (yrs), mean ± SD	45.1 ± 12.2	45.2 ± 12.0	44.8 ± 12.7	-
Male (%)	494 (63%)	326 (63%)	167 (63%)	-
Weight (kg), mean ± SD	92.6 ± 22.3	94.3 ± 22.4	89.0 ± 21.7	-
Biologic naïve, n (%)	583 (74%)	392 (75%)	191 (73%)	-
Immunosuppressant co-therapy, n (%)	238 (30%)	156 (30%)	82 (31%)	-
Psoriatic arthritis, n (%)	137 (26%)	85/349 (24%)	52/171 (30%)	264
Baseline PASI, mean ± SD	14.0 ± 7.3	14.3 ± 7.2	13.5 ± 7.5	93
Age at psoriasis onset, mean ± SD	22.0 ± 12.3	22.0 ± 12.3	22.0 ± 12.4	166
HLA-C*06:02 carrier status (%)	403 (51%)	264 (51%)	139 (53%)	-

Table E2. Baseline characteristics of 232 individuals sampled within 6 months of starting first course of adalimumab (replication cohort). ADA+: detectable ADA, ADA-: undetectable ADA.

	All	ADA+	ADA-
Number	232	150 (65%)	82 (35%)
Age (yrs), mean ± SD	43.4 ± 13.0	43.6 ± 12.8	43.0 ± 13.5
Male (%)	110 (47%)	72 (48%)	38 (46%)
Weight (kg), mean ± SD	89.5 ± 22.2	91.4 ± 23.2	86.2 ± 20.2
Biologic naïve (%)	189 (81%)	123 (82%)	66 (80%)
Immunosuppressant co-therapy (%)	54 (23%)	32 (21%)	22 (27%)

Table E3. Baseline characteristics of 716 individuals with clinical data but no ADA status available, on first course of adalimumab (adalimumab treatment duration cohort). ADA+: detectable ADA, ADA-: undetectable ADA.

	All	Terminated adalimumab due to inefficacy	Remainder of cohort
Number	716	145 (20%)	571 (80%)
Age (yrs), mean ± SD	45.4 ± 12.6	44.7 ± 12.7	45.6 ± 12.6
Male (%)	412 (58%)	86 (59%)	326 (57%)
Weight (kg), mean ± SD	90.4 ± 20.6	95.9 ± 21.4	89.0 ± 20.2
Biologic naïve (%)	515 (72%)	101 (70%)	414 (73%)
Immunosuppressant co-therapy (%)	277 (39%)	60 (41%)	217 (38%)

Table E4. Sensitivity analyses to assess robustness of the HLA-DRB1 joint model to potential confounders (discovery cohort: individuals sampled 6-36 months after treatment initiation, n=784 unless stated otherwise in first column). 9W: presence of tryptophan at position 9. 71K: presence of lysine at position 71.

Sensitivity analysis	Model term	OR	95% CI	P value
Excluding 238/784 individuals on immunosuppressant co-therapy (n=546)	9W	0.40	0.27-0.60	3.05×10^{-7}
	71K	0.46	0.30-0.68	1.43×10^{-4}
Increasing the threshold for ADA positivity from 30 AU (detectable) to 100 AU	9W	0.46	0.35-0.59	1.34×10^{-9}
	71K	0.56	0.42-0.74	6.61×10^{-5}
Including HLA-C*06:02 status as a covariate	9W	0.33	0.24-0.45	3.17×10^{-12}
	71K	0.40	0.28-0.55	6.69×10^{-8}
Including sample timing as a covariate	9W	0.35	0.26-0.48	2.40×10^{-11}
	71K	0.41	0.29-0.57	2.30×10^{-7}
Including psoriatic arthritis status as a covariate (n=520)	9W	0.31	0.21-0.46	4.08×10^{-9}
	71K	0.34	0.22-0.51	5.51×10^{-7}
Including age as a covariate	9W	0.34	0.25-0.45	1.94×10^{-12}
	71K	0.39	0.28-0.55	5.77×10^{-8}
Including age of psoriasis onset as a covariate (n=618)	9W	0.33	0.23-0.47	1.36×10^{-9}
	71K	0.36	0.24-0.52	1.56×10^{-7}
Including gender as a covariate	9W	0.34	0.25-0.45	1.87×10^{-12}
	71K	0.39	0.28-0.45	6.21×10^{-8}
Including baseline PASI as a covariate (n=691)	9W	0.33	0.24-0.45	3.17×10^{-11}
	71K	0.38	0.26-0.55	3.19×10^{-7}
Including all covariates (n=441)	9W	0.29	0.18-0.45	1.19×10^{-7}
	71K	0.32	0.19-0.51	3.88×10^{-6}

Table E5. Summary results for marginal association tests of ADA development for each imputed HLA amino acid (discovery cohort: individuals sampled 6-36 months after treatment initiation, n=784).

SNP	A1	A2	FRQ	INFO	OR	SE	P
AA_DRB1_9_32660121_W	P	A	0.4879	1.0632	0.5739	0.1085	3.09E-07
AA_DRB1_11_32660115_SVD	P	A	0.5121	1.0632	1.7426	0.1085	3.09E-07
AA_DRB1_9_32660121_E	P	A	0.5013	1.0408	1.7392	0.1099	4.73E-07
AA_DRB1_11_32660115_SV	P	A	0.5013	1.0408	1.7392	0.1099	4.73E-07
AA_DRB1_140_32657458_A	P	A	0.4987	1.0408	0.575	0.1099	4.73E-07
AA_DRB1_13_32660109_SHG	P	A	0.4924	1.0304	1.7394	0.1107	5.69E-07
AA_DRB1_140_32657458_T	P	A	0.5005	1.0394	1.7324	0.1099	5.76E-07
AA_DRB1_11_32660115_SD	P	A	0.3208	0.9959	1.8013	0.1239	2.04E-06
AA_DRB1_-16_32665457_A	P	A	0.6811	0.9838	0.5563	0.1246	2.54E-06
AA_DRB1_-25_32665484_K	P	A	0.6811	0.9838	0.5563	0.1246	2.54E-06
AA_DRB1_10_32660118_Q	P	A	0.6811	0.984	0.5565	0.1246	2.57E-06
AA_DRB1_149_32657431_Q	P	A	0.69	0.9855	0.556	0.1257	3.02E-06
AA_DRB1_13_32660109_SG	P	A	0.31	0.9861	1.7971	0.1257	3.10E-06
AA_DRB1_10_32660118_Y	P	A	0.31	0.9861	1.7971	0.1257	3.10E-06
AA_DRB1_11_32660115_S	P	A	0.31	0.9861	1.7971	0.1257	3.10E-06
AA_DRB1_12_32660112_T	P	A	0.31	0.9861	1.7971	0.1257	3.10E-06
AA_DRB1_149_32657431_H	P	A	0.3092	0.986	1.7886	0.1257	3.74E-06
AA_DRB1_-16_32665457_V	P	A	0.2975	0.9878	1.8037	0.1276	3.77E-06
AA_DRB1_-25_32665484_R	P	A	0.2975	0.9878	1.8037	0.1276	3.77E-06
AA_DRB1_233_32656004_T	P	A	0.6923	0.9765	0.563	0.1262	5.35E-06
AA_DRB1_37_32660037_SF	P	A	0.5181	1.0645	0.617	0.1079	7.69E-06
AA_DRB1_233_32656004_R	P	A	0.2863	0.9854	1.7788	0.1292	8.25E-06
AA_DRB1_13_32660109_SH	P	A	0.4653	1.0321	1.6295	0.1104	9.76E-06
AA_DRB1_11_32660115_GL	P	A	0.3667	1.0031	0.6081	0.1127	1.02E-05
AA_DRB1_30_32660058_LC	P	A	0.3667	1.0031	0.6081	0.1127	1.02E-05
AA_DRB1_37_32660037_NY	P	A	0.4721	1.0545	1.6136	0.1086	1.06E-05
AA_DRB1_30_32660058_YHG	P	A	0.6244	0.9911	1.6353	0.113	1.36E-05
AA_DRB1_30_32660058_YGR	P	A	0.6235	0.994	1.6251	0.1127	1.65E-05
AA_DRB1_30_32660058_YHR	P	A	0.6224	0.9905	1.6203	0.1129	1.93E-05
AA_DRB1_11_32660115_SPV	P	A	0.6224	0.9905	1.6203	0.1129	1.93E-05
AA_DRB1_30_32660058_YG	P	A	0.6146	0.9815	1.6168	0.1131	2.15E-05
AA_DRB1_13_32660109_YF	P	A	0.3865	0.9779	0.6201	0.1134	2.49E-05
AA_DRB1_30_32660058_YH	P	A	0.6135	0.9779	1.6126	0.1134	2.49E-05
AA_DRB1_30_32660058_YR	P	A	0.6127	0.9807	1.6027	0.113	3.01E-05
AA_DRB1_13_32660109_S	P	A	0.2829	0.9639	1.719	0.1299	3.04E-05
AA_DRB1_28_32660064_E	P	A	0.3871	0.9824	0.6259	0.1129	3.30E-05
AA_DRB1_30_32660058_Y	P	A	0.6038	0.9676	1.5957	0.1135	3.84E-05
AA_DRB1_28_32660064_D	P	A	0.6021	0.9684	1.5771	0.1133	5.81E-05
AA_DRB1_67_32659947_F	P	A	0.0772	0.9848	2.4524	0.249	0.0003147
AA_DRB1_13_32660109_SRH	P	A	0.5865	0.9966	1.4761	0.1106	0.0004313
AA_DRB1_11_32660115_SPD	P	A	0.442	0.9733	1.4805	0.1124	0.0004795

AA_DRB1_11_32660115_PG	P	A	0.4062	1.0613	0.6882	0.1071	0.0004857
AA_DRB1_13_32660109_RY	P	A	0.4062	1.0613	0.6882	0.1071	0.0004857
AA_DRB1_58_32659974_E	P	A	0.0462	1.0051	3.3819	0.355	0.0005977
AA_DRB1_58_32659974_A	P	A	0.9538	1.0055	0.296	0.3548	0.0006014
AA_DRB1_-17_32665460_T	P	A	0.2028	1.0691	0.6497	0.1273	0.0007076
AA_DRB1_11_32660115_PL	P	A	0.2028	1.0691	0.6497	0.1273	0.0007076
AA_DRB1_37_32660037_S	P	A	0.2029	1.0685	0.6498	0.1273	0.0007104
AA_DRB1_11_32660115_SVL	P	A	0.5829	1.0399	1.4391	0.108	0.0007479
AA_DRB1_13_32660109_SRG	P	A	0.4311	0.9754	1.4583	0.1125	0.0007969
AA_DRB1_11_32660115_SP	P	A	0.4311	0.9754	1.4583	0.1125	0.0007971
AA_DRB1_96_32657590_QE	P	A	0.2117	1.0752	0.6663	0.125	0.001163
AA_DRB1_37_32660037_SL	P	A	0.2127	1.0625	0.6664	0.1258	0.001257
AA_DRB1_96_32657590_HY	P	A	0.7877	1.0733	1.4942	0.125	0.001318
AA_DRB1_11_32660115_SVG	P	A	0.7863	1.0471	1.4984	0.1266	0.001404
AA_DRB1_11_32660115_L	P	A	0.0816	1.0642	0.5627	0.1835	0.001732
AA_DRB1_30_32660058_C	P	A	0.0816	1.0642	0.5627	0.1835	0.001732
AA_DRB1_96_32657590_E	P	A	0.0816	1.0642	0.5627	0.1835	0.001732
AA_DRB1_-17_32665460_A	P	A	0.7758	1.0428	1.4782	0.1248	0.001736
AA_DRB1_32_32660052_H	P	A	0.2508	0.9676	1.5163	0.133	0.001745
AA_DRB1_96_32657590_Ex	P	A	0.0822	1.0615	0.5694	0.1832	0.002107
AA_DRB1_13_32660109_RF	P	A	0.2226	1.0527	0.6829	0.1245	0.00219
AA_DRB1_37_32660037_YL	P	A	0.2691	0.9885	1.4708	0.1276	0.002504
AA_DRB1_74_32659926_LE	P	A	0.0706	0.9328	2.1108	0.2509	0.0029
AA_DRB1_37_32660037_Y	P	A	0.2593	0.9812	1.4664	0.1295	0.003122
AA_DRB1_30_32660058_CR	P	A	0.0906	1.0707	0.6049	0.1747	0.004012
AA_DQB1_130_32737898_Q	P	A	0.0226	1.0263	8.4443	0.7431	0.004089
AA_DQB1_86_32740579_G	P	A	0.0226	1.0265	8.4385	0.7431	0.004104
AA_DRB1_13_32660109_SHF	P	A	0.5667	1.0696	1.3516	0.1056	0.004334
AA_DQB1_130_32737898_R	P	A	0.9762	1.0236	0.1751	0.6131	0.004482
AA_DQB1_86_32740579_EA	P	A	0.9762	1.0237	0.1751	0.6131	0.004491
AA_DRB1_71_32659935_E	P	A	0.0946	0.9518	1.8178	0.2106	0.004537
AA_DQA1_47_32717191_K	P	A	0.2844	0.9793	0.715	0.1197	0.005053
AA_DQA1_52_32717206_H	P	A	0.2844	0.9793	0.715	0.1197	0.005053
AA_DQA1_54_32717212_L	P	A	0.2844	0.9793	0.715	0.1197	0.005053
AA_DRB1_30_32660058_CH	P	A	0.0906	1.0619	0.6112	0.1758	0.005089
AA_DRB1_11_32660115_G	P	A	0.2851	0.983	0.7162	0.1194	0.005158
AA_DRB1_13_32660109_Y	P	A	0.2851	0.983	0.7162	0.1194	0.005158
AA_DRB1_14_32660106_K	P	A	0.2851	0.983	0.7162	0.1194	0.005158
AA_DRB1_25_32660073_Q	P	A	0.2851	0.983	0.7162	0.1194	0.005158
AA_DRB1_30_32660058_L	P	A	0.2851	0.983	0.7162	0.1194	0.005158
AA_DRB1_74_32659926_Q	P	A	0.2851	0.983	0.7162	0.1194	0.005159
AA_DRB1_57_32659977_DV	P	A	0.9416	0.9829	0.4737	0.2681	0.005328
AA_DRB1_13_32660109_SFG	P	A	0.4114	1.0123	1.3627	0.1113	0.005413
AA_DRB1_13_32660109_SR	P	A	0.4041	0.9735	1.3675	0.1133	0.005722
AA_DRB1_30_32660058_CG	P	A	0.0925	1.05	0.6165	0.1751	0.00574
AA_DRB1_31_32660055_I	P	A	0.0925	1.05	0.6165	0.1751	0.00574
AA_DRB1_11_32660115_LD	P	A	0.0925	1.05	0.6165	0.1751	0.005742

AA_DRB1_11_32660115_SLD	P	A	0.4024	1.0107	1.3564	0.1118	0.006402
AA_DRB1_30_32660058_LR	P	A	0.294	0.9645	0.7217	0.1197	0.006423
AA_DQA1_52_32717206_R	P	A	0.4024	1.0206	1.3527	0.1111	0.006531
AA_DQA1_47_32717191_RK	P	A	0.5976	1.0206	0.7393	0.1111	0.006531
AA_DRB1_30_32660058_LH	P	A	0.2948	0.9745	0.7281	0.1188	0.007551
AA_DRB1_30_32660058_LG	P	A	0.2959	0.9715	0.7315	0.119	0.00861
AA_DRB1_4_32665400_Q	P	A	0.2959	0.9715	0.7315	0.119	0.00861
AA_DRB1_11_32660115_GD	P	A	0.2959	0.9715	0.7315	0.119	0.008612
AA_DRB1_78_32659914_V	P	A	0.2959	0.9715	0.7316	0.119	0.008615
AA_DRB1_30_32660058_YLG	P	A	0.8997	1.0676	1.5469	0.1676	0.009263
AA_DRB1_26_32660070_L	P	A	0.1003	1.0678	0.6465	0.1676	0.009268
AA_DRB1_30_32660058_YCG	P	A	0.6962	0.9557	1.3634	0.1191	0.00927
AA_DRB1_11_32660115_SL	P	A	0.3916	1.0041	1.3404	0.1126	0.009276
AA_DQB1_-17_32742316_A	P	A	0.9777	1.0182	0.2264	0.5723	0.009455
AA_DRB1_181_32657335_M	P	A	0.3048	0.9527	0.7368	0.1194	0.01052
AA_DRB1_30_32660058_YCH	P	A	0.6952	0.9527	1.3572	0.1194	0.01052
AA_DRB1_13_32660109_F	P	A	0.1014	1.0549	0.6523	0.1681	0.01102
AA_DRB1_30_32660058_YLH	P	A	0.8986	1.0549	1.533	0.1681	0.01102
AA_DRB1_31_32660055_F	P	A	0.8986	1.0549	1.533	0.1681	0.01102
AA_DRB1_37_32660037_NL	P	A	0.2226	0.9756	1.4137	0.1372	0.01165
AA_DRB1_4_32665400_R	P	A	0.7023	0.9691	1.3495	0.119	0.01174
AA_DRB1_30_32660058_YCR	P	A	0.6943	0.9625	1.3459	0.1185	0.01221
AA_DRB1_60_32659968_S	P	A	0.3057	0.9625	0.743	0.1185	0.01222
AA_DRB1_57_32659977_V	P	A	0.3057	0.9625	0.743	0.1185	0.01223
AA_DQA1_34_32717152_E	P	A	0.5944	0.9842	0.7555	0.1124	0.01259
AA_DRB1_30_32660058_YLR	P	A	0.8977	1.0484	1.5211	0.1683	0.01271
AA_DRB1_37_32660037_N	P	A	0.2128	0.9631	1.4114	0.1403	0.01405
AA_DRB1_73_32659929_G	P	A	0.4133	0.9528	0.7591	0.1123	0.01409
AA_DRB1_74_32659926_RQ	P	A	0.4133	0.9528	0.7591	0.1123	0.01409
AA_DRB1_74_32659926_E	P	A	0.0563	0.9272	1.9661	0.2755	0.01413
AA_DRB1_30_32660058_YC	P	A	0.6854	0.9433	1.3367	0.119	0.01475
AA_DPB1_194_33161555_Q	P	A	0.0517	1.1277	0.5866	0.2201	0.01538
AA_DRB1_47_32660007_F	P	A	0.3693	0.9963	1.3124	0.114	0.01711
AA_DRB1_13_32660109_SHY	P	A	0.7503	1.0131	1.3268	0.1227	0.0212
AA_DRB1_30_32660058_YL	P	A	0.8888	1.0514	1.4517	0.1623	0.02167
AA_DRB1_74_32659926_QL	P	A	0.2993	1.0225	0.7684	0.1154	0.02239
AA_DQA1_40_32717170_G	P	A	0.2085	0.9529	1.3662	0.1404	0.02629
AA_DQA1_47_32717191_C	P	A	0.2085	0.9529	1.3662	0.1404	0.02629
AA_DQA1_50_32717200_V	P	A	0.2085	0.9529	1.3662	0.1404	0.02629
AA_DQA1_51_32717203_L	P	A	0.2085	0.9529	1.3662	0.1404	0.02629
AA_DQA1_53_32717209_Q	P	A	0.2085	0.9529	1.3662	0.1404	0.02629
AA_DRB1_13_32660109_SF	P	A	0.3843	1.0022	1.2815	0.1127	0.02775
AA_DRB1_67_32659947_I	P	A	0.5075	1.0309	0.7952	0.1067	0.0318
AA_DRB1_181_32657335_T	P	A	0.6838	0.9846	1.2832	0.1162	0.03196
AA_DQB1_77_32740606_R	P	A	0.3989	1.0533	0.7975	0.1072	0.03472
AA_DRB1_71_32659935_KA	P	A	0.37	1.041	0.7967	0.109	0.03707
AA_DQB1_71_32740624_KA	P	A	0.3977	1.0522	0.8004	0.1072	0.03787

AA_DRB1_74_32659926_AQ	P	A	0.8012	0.9924	0.7475	0.1406	0.03855
AA_DRB1_70_32659938_R	P	A	0.0498	0.9721	1.7723	0.279	0.04025
AA_DRB1_13_32660109_YG	P	A	0.3121	1.0132	0.7924	0.1146	0.04228
AA_DPB1_55_33156577_E	P	A	0.0321	0.9229	2.1246	0.3727	0.0432
AA_DRB1_96_32657590_Hx	P	A	0.606	0.995	1.2499	0.1103	0.04326
AA_DRB1_11_32660115_SGD	P	A	0.6059	0.9957	1.2491	0.1103	0.04373
AA_DRB1_74_32659926_AE	P	A	0.5724	0.9823	1.2473	0.11	0.0445
AA_DRB1_37_32660037_F	P	A	0.3152	0.9713	0.792	0.1169	0.04599
AA_DRB1_96_32657590_H	P	A	0.6054	0.9953	1.2453	0.1103	0.04673
AA_DRB1_57_32659977_A	P	A	0.0301	0.9655	2.0706	0.3678	0.04782
AA_DRB1_60_32659968_H	P	A	0.0301	0.9654	2.0705	0.3678	0.04782

Table E6. Summary results for marginal association tests of ADA development for each classical HLA allele (discovery cohort: individuals sampled 6-36 months after treatment initiation, n=784).

SNP	A1	A2	FRQ	INFO	OR	SE	P
HLA_DRB1_11	P	A	0.0462	1.0054	3.3793	0.3548	0.0006003
HLA_DRB1_01	P	A	0.0816	1.0642	0.5628	0.1835	0.001733
HLA_DRB1_1302	P	A	0.0223	1.0356	8.0544	0.7326	0.004402
HLA_DQA1_02	P	A	0.2844	0.9793	0.715	0.1197	0.005053
HLA_DQA1_0201	P	A	0.2844	0.9793	0.715	0.1197	0.005053
HLA_DRB1_07	P	A	0.2851	0.983	0.7162	0.1194	0.005159
HLA_DRB1_0701	P	A	0.2851	0.983	0.7162	0.1194	0.005159
HLA_DQB1_0501	P	A	0.0849	1.0867	0.6136	0.1785	0.006207
HLA_DRB1_13	P	A	0.0798	0.9571	1.8167	0.2276	0.008706
HLA_DPB1_01	P	A	0.0517	1.1277	0.5865	0.2201	0.01537
HLA_DPB1_0101	P	A	0.0517	1.1277	0.5865	0.2201	0.01537
HLA_DQB1_0604	P	A	0.0167	1.0537	5.7394	0.7483	0.01954
HLA_DRB1_0101	P	A	0.0599	1.0835	0.6245	0.2094	0.02455
HLA_DRB1_14	P	A	0.0318	0.9487	2.2516	0.3683	0.02754
HLA_DQB1_0302	P	A	0.0932	0.957	1.5338	0.2026	0.03475
HLA_DQB1_0503	P	A	0.031	0.9626	2.1587	0.3666	0.03583
HLA_DRB1_1501	P	A	0.1091	1.113	0.7317	0.1591	0.04953

Table E7. Classical HLA-DRB1 alleles ordered by associated risk of developing ADA. Corresponding amino acids at positions 9 and 71 are shown (discovery cohort: individuals sampled 6-36 months after treatment initiation, n=784). †denotes classical HLA-DRB1 alleles harbouring protective residues at either position 9 or position 71. W: tryptophan, E: glutamic acid, K: lysine, R: arginine.

	Freq	OR	SE	P value	Position 9	Position 71
**HLA-DRB1*01†**	0.0816	0.5628	0.1835	0.001733	W	R
**HLA-DRB1*07†**	0.2851	0.7162	0.1194	0.005159	W	R
**HLA-DRB1*15†**	0.1130	0.7761	0.1582	0.109	W	A
**HLA-DRB1*03†**	0.1282	1.0375	0.1652	0.8238	E	K
**HLA-DRB1*16†**	0.0082	1.1128	0.6151	0.8621	W	R
**HLA-DRB1*04†**	0.1824	1.1579	0.1425	0.3035	E	K
**HLA-DRB1*10**	0.0089	1.2430	0.6008	0.7173	E	R
**HLA-DRB1*12**	0.0098	1.3287	0.5746	0.6209	E	R
**HLA-DRB1*09**	0.0108	1.5096	0.5809	0.4784	K	R
**HLA-DRB1*13**	0.0798	1.8167	0.2276	0.008706	E	E
**HLA-DRB1*14**	0.0318	2.2516	0.3683	0.02754	E	R
**HLA-DRB1*08**	0.0142	2.5133	0.5674	0.1043	E	R
**HLA-DRB1*11**	0.0462	3.3793	0.3548	0.0006003	E	R
